# Understanding of researcher behavior is required to improve data reliability

**DOI:** 10.1093/gigascience/giz017

**Published:** 2019-01-31

**Authors:** Mark N Wass, Larry Ray, Martin Michaelis

**Affiliations:** 1 Industrial Biotechnology Centre and School of Biosciences, University of Kent, Canterbury, CT2 7NJ, UK; 2 School of Social Policy, Sociology and Social Research, University of Kent, Canterbury, CT2 7NJ, UK

**Keywords:** reproducibility crisis, replication crisis, data reliability, bias, publication bias, meta-research

## Abstract

**Background:**

A lack of data reproducibility (“reproducibility crisis”) has been extensively debated across many academic disciplines.

**Results:**

Although a reproducibility crisis is widely perceived, conclusive data on the scale of the problem and the underlying reasons are largely lacking. The debate is primarily focused on methodological issues. However, examples such as the use of misidentified cell lines illustrate that the availability of reliable methods does not guarantee good practice. Moreover, research is often characterized by a lack of established methods. Despite the crucial importance of researcher conduct, research and conclusive data on the determinants of researcher behavior are widely missing.

**Conclusion:**

Meta-research that establishes an understanding of the factors that determine researcher behavior is urgently needed. This knowledge can then be used to implement and iteratively improve measures that incentivize researchers to apply the highest standards, resulting in high-quality data.

## Background

**Figure 1:**
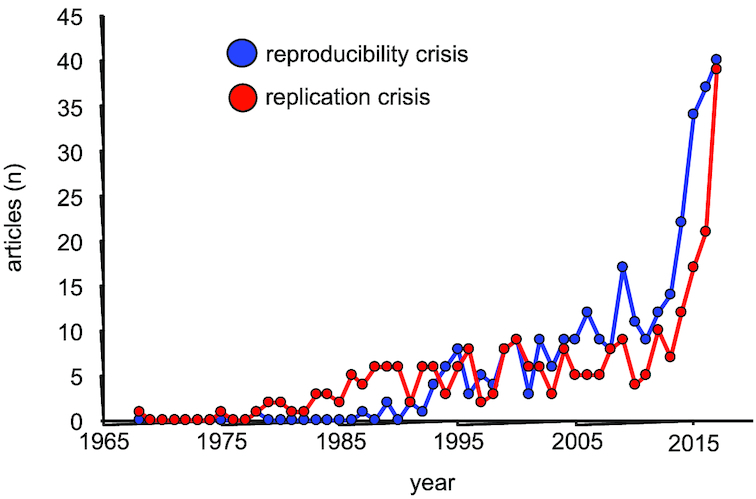
Number of articles that are identified by the search terms “replication crisis” (red) or “reproducibility crisis” (blue) per year from 1965 to 2017 in PubMed (13], data accessed on 12 January 2018).

A lack of data reproducibility (“reproducibility crisis”) is debated across many medical and scientific disciplines [1–12]. It seems to receive increasing attention, as demonstrated by the increase in articles indexed in PubMed [13] related to the terms “reproducibility crisis” and “replication crisis” (Fig. 1). This finding is in agreement with another recent analysis that indicated a rapidly increasing number of scientific articles within a “crisis narrative” [14]. Factors suggested to affect reproducibility include (a lack of) methodological standards, (unconscious) bias, pressure related to the need to attract grants and publish in “high-impact” journals, and publication bias favoring the publication of novel (“positive”) findings and discouraging the publication of confirmatory findings and “negative” results [3, 11, 15–22]. Some authors argue that a high proportion (up to 90%) of research money is wasted [2–7]. However, this very pessimistic view may not be widely shared. Other authors argue that the crisis narrative is exaggerated and that periods of self-correction and self-improvement are an immanent feature of scientific research [14, 23]. Nevertheless, the perception of a reproducibility crisis seems to be common among researchers. In two *Nature* surveys, the majority of respondents (52% of 1,576 respondents, 86% of 480 respondents) agreed that a reproducibility crisis exists [24, 25].

## Results

### Scale of crisis remains unclear

Despite the high visibility of the issue, systematic research and in turn conclusive evidence on the scale of a potential reproducibility crisis are lacking. In a survey among faculty and trainees at the MD Anderson Cancer Center, about 50% of the participants reported that they had failed to reproduce published data at least once [26]. Similarly, in a *Nature* survey >70% of the 1,576 respondents stated that they had been unable to reproduce data at least once [24]. However, systematic data that would enable the reliable quantification of the issue are lacking.

In the Reproducibility Project: Cancer Biology by the Center for Open Science [27] and Science Exchange [28], findings from 29 high-profile scientific publications will be independently replicated [29–31]. To date, the results of 11 replication studies have been reported. Important parts of the original paper could be reproduced in four studies [32–35]. The results from two replication studies could not be interpreted [36, 37], and two studies failed to replicate the original findings [38, 39]. In three further reports, some parts of the original studies were reproduced while others were not [40–42] (Table 1).

**Table 1:** Replication studies performed as part of the Replication Project: Cancer Biology [30], presented according to the outcome as interpreted in the “Editors” Summary

First author	Title
*Editors' Summary: This Replication Study has reproduced important parts of the original paper*.	
Irawati Kandela	Replication Study: Discovery and preclinical validation of drug indications using compendia of public gene expression data [32]^1^
Fraser Aird	Replication Study: BET bromodomain inhibition as a therapeutic strategy to target c-Myc [31]
Xiaochuan Shan	Replication Study: Inhibition of BET recruitment to chromatin as an effective treatment for MLL-fusion leukaemia [33]
Megan Reed Showalter	Replication Study: The common feature of leukemia-associated IDH1 and IDH2 mutations is a neomorphic enzyme activity converting alpha-ketoglutarate to 2-hydroxyglutarate [34]
*Editors' Summary: This Replication Study has reproduced important parts of the original paper, but it also contains results that are not consistent with some parts of the original paper*.	
L Michelle Lewis	Replication Study: Transcriptional amplification in tumor cells with elevated c-Myc [39]
*Editors' Summary: This Replication Study has reproduced some parts of the original paper but other parts could not be interpreted*.	
John P Vanden Heuvel	Replication Study: Systematic identification of genomic markers of drug sensitivity in cancer cells [40]
*Editors' Summary: The results in this Replication Study could not be interpreted*.	
Stephen K Horrigan	Replication Study: Melanoma genome sequencing reveals frequent PREX2 mutations [36]
Stephen K Horrigan	Replication Study: The CD47-signal regulatory protein alpha (SIRPa) interaction is a therapeutic target for human solid tumors [35]
*Editors' Summary: This Replication Study has reproduced some parts of the original paper but it also contains results that are not consistent with other parts of the original paper*.	
Kathryn Eaton	Replication Study: Intestinal inflammation targets cancer-inducing activity of the microbiota [41]
*Editors' Summary: This Replication Study did not reproduce those experiments in the original paper that it attempted to reproduce*.	
Christine Mantis	Replication Study: Coadministration of a tumor-penetrating peptide enhances the efficacy of cancer drugs [37]
John Repass	Replication Study: *Fusobacterium nucleatum* infection is prevalent in human colorectal carcinoma [38]

^1^Number in the reference list.

Psychological studies also seem to vary with regard to replication success. Very low levels of reproducibility have been reported in some cases [43, 44]. A study by the Open Science Collaboration reported the successful replication of 39 of 100 psychological studies [9]. However, other studies replicated a majority of the analyzed effects [45] or confirmed previous findings [46, 47]. A dataset provided a qualitative list of 54 replication attempts of implicit Theory of Mind paradigms based on a survey [48]. Twenty-six studies (48%) were successfully replicated, 15 studies (28%) were partially replicated, and 13 studies (24%) were not successfully replicated [48].

In the clinical research field, an analysis of follow-up publications of 49 original clinical research studies that had been published between 1990 and 2003 and had each acquired more than 1000 citations revealed that 7 (16%) were not confirmed by subsequent studies, 7 (16%) had reported stronger effects than those found in subsequent studies, 20 (44%) were successfully replicated, and for 11 (24%) follow-up data were not available [1]. Another study compared the results from a limited number of initial clinical studies and respective follow-up studies. It concluded that less than 50% of the investigated studies reported reproducible effects [49]. However, it is not clear how representative the data are.

Notably, reproducibility data have also been reported in articles other than original research articles. For example, researchers from drug companies reported that only 6 out of 53 studies (11%) [5] or 16 out of 67 studies (24%) [3] had been successfully reproduced. However, these data were published as a Comment [5] and a Correspondence [3] without presentation of detailed data. Hence, the exact nature of the investigations and the criteria for reproducibility remain elusive.

Taken together, there are anecdotal reports of data irreproducibility. However, the actual scale of the issue remains unclear due to a lack of systematic data. Most replication attempts focus on highly cited early-stage studies. This may not adequately reflect the general reproducibility of research findings. A meta-assessment of bias in the sciences observed a significant risk of small, early, and highly cited studies to overestimate effects [50]. Further, failed and successful replication attempts would need to be systematically analyzed together to provide meaningful insights. However, such studies are not available. A psychology study estimated that only about 1% of studies are subject to replication attempts [51].

Some studies have investigated the extent to which researchers may be able to estimate the reproducibility of data, but conclusive evidence is still missing. Individual cancer researchers were not able to predict accurately whether studies would be reproducible in the Reproducibility Project: Cancer Biology [29, 52]. However, studies from the social and psychological sciences suggested that the “wisdom of the crowd” of researchers in the respective fields predicts the reproducibility with higher accuracy than expected by chance [53, 54].

The determination of the scale of the problem may be further complicated by the absence of clear criteria that define the successful or unsuccessful repetition of a study. For example, two large pharmacogenomics screens in cancer cell lines [55, 56] provoked a dispute on the consistency of the data, which resulted in at least 10 research articles and letters [57–66]. Six of these contributions reported discrepancies between the datasets, while four reported consistency. All six contributions that reported discrepancies were published by the same research group, whereas the articles reporting consistency were published by four different research groups (Table 2). The dispute does not appear to have been resolved. This illustrates that the criteria for reproducibility may differ significantly among researchers. In this context, a modeling study from the psychology field suggests that the criteria for reproducibility may sometimes be interpreted in an unrealistically strict fashion [67].

**Table 2:** Articles contributing to a dispute on the consistence of the data derived from two large pharmacogenomic screens [51, 52]

First author	Title
*In favor of consistence*	
JP Mpindi	Consistency in drug response profiling [57]
M Bouhaddou	Drug response consistency in CCLE and CGP [55]
P Geeleher	Consistency in large pharmacogenomic studies [56]
Cancer Cell Line Encyclopedia Consortium; Genomics of Drug Sensitivity in Cancer Consortium.	Pharmacogenomic agreement between two cancer cell line data sets [54]
*In dispute of consistence*	
Z. Safikhani	Revisiting inconsistency in large pharmacogenomic studies [62]
Z. Safikhani	Safikhani et al. reply [58]
Z. Safikhani	Safikhani et al. reply [59]
Z. Safikhani	Safikhani et al. reply [60]
Z. Safikhani	Assessment of pharmacogenomic agreement [61]
B Haibe-Kains	Inconsistency in large pharmacogenomic studies [53]

### Initiatives focus on methodology, data transparency, researcher training, and institutional standards

The issue of limited reproducibility has also been recognized by research funders and scientific journals [68, 69]. For example, the UK funders Medical Research Council, Academy of Medical Sciences, Wellcome Trust, and Biotechnology and Biological Sciences Research Council published a common report on data reproducibility [70], and the World Economic Forum established a Code of Ethics for Researchers [71]. Initiatives to improve data reproducibility typically focus on methodological issues and data transparency. Journals have also tried to address the problem with publishers including the Nature Publishing Group and EMBO Press introducing “publication checklists” [see, e.g., 25, 72, 73]. *Nature* also published a special collection on reproducibility in 2013 [74]. Moreover, researcher training and institutional standards including quality management systems have been suggested [8, 69, 75, 76].

### Impact of suggested measures is not clear

However, limited data are available on the impact of the suggested measures to improve data quality and reproducibility. There are recent reports on shortcomings in data sharing in metabolomic studies [77] and limited adherence to animal reporting guidelines in Korea [78]. A survey reported that psychologists were open to changes to data collection, reporting, and publication practices but less positive about mandatory conditions of publication [79]. Forty-nine percent of 480 respondents (out of 5,375 researchers who had published in a Nature Publishing Group journal between July 2016 and March 2017 and who had received the survey) of a Nature Publishing Group survey felt that the checklist had improved the quality of research published in Nature Publishing Group journals [25]. However, it remains unclear if this cohort is representative. One study suggested that reporting of randomization, blinding, and sample-size estimation in animal experiments had improved in the journal *Nature* in response to the introduction of the publication checklist based on a comparison of articles published in *Nature* and *Cell* from 2013 to 2015 [80]. A preprint posted on bioRxiv also concluded that the introduction of a checklist by *Nature* had improved study design and the transparency of data [81], but data indicating whether this translated into improved reproducibility are not yet available.

Many authors argue in favor of the standardization of methods and higher requirements for experimental design [5, 18–21, 82–84]. In the area of drug discovery, clear requirements for the generation of reproducible data have been suggested [see, e.g., 19, 21, 22, 85]. However, data on the implementation of such measures and their efficacy with regard to improved reproducibility are not available. In addition, there is not yet a consensus on the correct methodological approach to achieve high reproducibility. In animal experiments, batch-to-batch variation was described even under highly standardized conditions in the same lab [86]. In this context, experiment heterogenization and a multi-laboratory design were suggested to produce more reliable data [86–90] instead of increased standardization. Notably, standardization is only an option if the appropriate procedure that delivers correct results is known. Otherwise, a standardized approach may produce flawed results with high reproducibility.

### The availability of appropriate methods does not ensure good practice

Despite the focus of the debate on research methodology and reporting guidelines, it remains unclear whether (and if, yes, to what extent) a lack of reproducibility may be caused by a lack of (knowledge of) appropriate methods and to what extent the significance of data can be improved by tighter guidelines and standardization.

With regard to the use of appropriate methodologies, cell line misidentification has been an area of concern since the first cell lines were established [91, 92]. Although short tandem repeat analysis has been available and promoted as a reliable authentication method since at least 2001 [93], very recent articles continue to demonstrate that the use of misidentified cell lines remains an issue [94–96]. Similar issues have been reported on the use of antibodies that lack specificity [97–100].

A meta-analysis considering articles published over a 60-year period indicated that the statistical power of behavioral sciences studies has not increased, although the need to increase the statistical power was repeatedly discussed and demonstrated [101]. Hence, the availability of suitable and reliable methods is not sufficient to guarantee their appropriate and consequent use. Additionally, it is often a characteristic of research that both experiments are performed and methodologies are used for the first time. Consequently, researcher conduct and the research culture are critical to ensure the highest possible reliability of data. Accordingly, 82% of the 480 Nature Publishing Group survey respondents felt that researchers have the greatest capacity to improve the reproducibility of published work. In addition, 58% thought that individual researchers and 24% thought that laboratory heads were in a crucial position to improve data reliability [25]. Hence, more focus and effort need to be invested to understand how researchers report and present their data and why they do what they do. In this context, 66% of the respondents stated “selective reporting” as a factor that contributes to limited reproducibility [25].

### Role of the incentive system

Research is performed in a competitive environment. Researchers’ careers are driven by publications in as highly prestigious research journals as possible to gain visibility and attract research funding [19, 69, 102]. This requires the presentation of novel, significant findings, which incentivizes the publication of “positive” findings and discourages the publication of “negative” findings. This may also incentivize smaller (potentially underpowered) studies because they are more likely to produce significant results than larger studies [19, 102]. A modeling study indicated that the best strategy to produce significant findings and optimize research output is to perform small studies that only have 10%–40% statistical power, which would result in half of the studies reporting false-positive findings [103]. Further, modeling studies suggested that pressure to produce a high number of outputs with a focus on novel findings and positive results undermines the rigorousness of science because it leads to a higher proportion of false positives [101, 104]. Accordingly, early, highly cited studies seem to be more likely to present exaggerated findings [50]. However, it remains unclear if (and if, yes, to what extent) such strategies significantly affect researcher conduct (consciously or subconsciously) and data reproducibility.

### Contribution of publication bias

A focus on “positive” results also favors “publication bias,” i.e., “positive” results are more likely to be published than “negative” findings. Hence, the available literature does not appropriately represent the totality of experiments that have been performed because many “negative” results remain unpublished (“file drawer problem”). Additionally, “positive” findings are more likely to be published in prestigious journals than "negative" findings [18, 19, 105].

One study reported the overestimation of the importance of anticipated prognostic factors in various types of cancer due to publication bias [106]. A follow-up study, which investigated 1,915 research articles on prognostic markers in cancer, found that >90% of studies reported positive prognostic correlations [107]. Less than 1.5% of the investigated articles provided purely “negative” data. Where “negative” findings were presented, this typically happened in the context of other significant correlations (“positive” findings), or the authors followed up on non-significant trends and tried to defend the importance of the investigated markers despite the lack of significance [107]. This illustrates that negative results are not commonly published. The evaluation of meta-analyses on cancer biomarkers and the analysis of animal studies on stroke and neurological diseases also suggested a bias towards the publication of “positive results” [108–110].

Further, a similar publication bias was reported for both clinical trials [111, 112] and psychological studies [113, 114]. A survey-based dataset listed replication attempts of implicit Theory of Mind paradigms. A total of 28 out of the 54 studies, which were reported by the survey respondents, had been published in peer-reviewed scientific journals [48]. The vast majority of published studies (23/82%) reported successful replications. Four studies (14%) reported partial replications, and only one study (4%) reported a failed replication attempt. In sharp contrast, only 3 of the 26 unpublished replication studies (12%) reported successful replication. Eleven unpublished studies (42%) reported partial replication, while 12 unpublished studies (46%) were unsuccessful replication attempts [48]. Accordingly, a large analysis using US data concluded that there is a general publication bias towards the publication of “positive” results across the academic disciplines [115]. This bias seems to be more pronounced when fewer results are characterized by exact quantitative data [116]. Notably, this topic becomes complicated by findings that suggest that meta-research on publication bias may itself be subject to publication bias [117]. Taken together, there is convincing evidence that a bias favoring the publication of "positive" findings exists and that it may affect the reliability of publicly available data. However, the scale of the impact is not clear.

### Further determinants of researcher conduct and the impact on data reproducibility are unclear

Researcher conduct defines the reliability of findings beyond publication bias. This is highly relevant as original research is typically defined by a significant level of novelty in the absence of established standards. Findings are often made using novel (combinations of) approaches together with (novel) model systems and/or (novel) data for the first time, i.e., before tested and standardized approaches are available. It is fair to think that the incentives provided in a research environment substantially influence researcher behavior. A substantial meta-analysis based on data from 18 surveys concluded that a pooled weighted estimate of 1.97% (crude unweighted mean: 2.59%) of the respondents admitted to have fabricated, falsified, or modified data or results at least once, and 14.12% (crude unweighted mean: 16.66%) reported to personally know of a colleague who had done so [118]. Hence, there is evidence of questionable research practices, but the actual extent, the influence of the research environment and its incentives, and the concrete effect on data reliability remain elusive.

Studies that investigated researcher (mis)conduct in response to the pressures and incentives of the research environment are rare. A survey analyzing the answers from 3,247 early- and mid-career scientists suggested that a feeling of injustice may contribute to questionable research practices, which may affect reproducibility [119, 120]. Focus group discussions involving 51 scientists from research universities revealed that the pressure to produce outputs also promotes questionable research practices [121], which may affect reproducibility. In a survey of 315 Flemish biomedical scientists, 15% of the respondents admitted that they had fabricated, falsified, plagiarized, or manipulated data in the past three years, and 72% rated the publication pressure as “too high” [122]. A follow-up qualitative focus group interview study among Dutch biomedical researchers suggested that the current publication culture leads to questionable research practices among junior and senior biomedical scientists [123]. Hence, there is some initial evidence that the pressure associated with a highly competitive environment affects researcher conduct, which in turn affects the reliability and reproducibility of data. Again, however, the actual scale and impact on data reliability remain elusive.

## Conclusions

A reproducibility crisis is widely recognized among researchers from many different fields [24, 25]. There is no shortage of suggestions on how data reproducibility could be improved [5, 8, 11, 15–19, 21, 22, 69, 72, 73, 82–85, 87, 97, 113], but quantitative data on the subject (including the scale of the problem) are largely missing. Currently, there is a strong focus on methodology. However, ongoing issues with the use of misidentified cell lines illustrate that problems may persist, despite effective standards being available. Further, it is in the nature of research to do things for the first time before established methods are available. Hence, data reliability is primarily defined by the conduct of researchers and their rigor and scrutiny in the acquisition, analysis, interpretation, and presentation of data.

Publication bias favors the publication of “positive” results. Moreover, there are initial indications that the high pressure associated with a competitive environment increases the preparedness of researchers to lower their ethical standards, but the available information remains scarce and the actual impact unclear. Hence, systematic (meta-)research is needed on the topic in order to quantify the issue and generate the knowledge that is necessary to improve data quality and reproducibility. Actual fraud seems to be rare and the exception [14]. Consequently, a major focus of meta-research on data reproducibility will need to be put on researcher behavior in areas that are not considered to be “fraud” but that still may affect the robustness of data. “Boundary work,” i.e., the ways researchers draw the boundaries between the permissible and the non-permissible [124], will be critical here. Only measures that are based on a detailed understanding of researchers' behavior and that are closely monitored for efficacy (and iteratively improved) will make it possible to amend our research system in a way that it provides the right incentives to ensure that researchers apply the highest possible standards and provide high-quality data.

## Availability of data and materials

All data are available in the manuscript.

## Competing Interest

There are no competing interests.

## Author contributions

All authors analyzed data, contributed to the writing of the article, and approved the final version.

## Supplementary Material

GIGA-D-18-00445_Original_Submission.pdf

GIGA-D-18-00445_Revision_1.pdf

Response_to_Reviewer_Comments_Original_Submission.pdf

Reviewer_1_Report_Original_Submission -- Timothy M. Errington, Ph.D. 12/6/2018 Reviewed
