# Supplementary material for: Understanding of researcher behavior is required to improve data reliability
Source: Gigascience. 2019 Jan 31;8(5):giz017. doi: 10.1093/gigascience/giz017 (PMC6528747; doi:10.1093/gigascience/giz017)
Supplement: GIGA-D-18-00445_Original_Submission.pdf [file giz017_giga-d-18-00445_original_submission.pdf]

# GigaScience

## Understanding of researcher behaviour is required to improve data reliability

--Manuscript Draft--

| | |
|---|---|
| **Manuscript Number:** | GIGA-D-18-00445 |
| **Full Title:** | Understanding of researcher behaviour is required to improve data reliability |
| **Article Type:** | Review |
| **Funding Information:** | |
| **Abstract:** | Background<br><br>A lack of data reproducibility (“reproducibility crisis”) has been extensively debated across many academic disciplines.<br><br>Main body<br><br>Although a reproducibility crisis is widely perceived, conclusive data on the scale of the problem and the underlying reasons are largely lacking. The debate is primarily focused on methodological issues. However, examples such as the use of misidentified cell lines illustrate that the availability of reliable methods does not guarantee good practice. Moreover, research is often characterised by a lack of established methods. Despite the crucial importance of researcher conduct, research and conclusive data on the determinants of researcher behaviour are widely missing.<br><br>Conclusion<br><br>Meta-research is urgently needed that establishes an understanding of the factors that determine researcher behaviour. This knowledge can then be used to implement and iteratively improve measures, which incentivise researchers to apply the highest standards resulting in high quality data. |
| **Corresponding Author:** | Martin Michaelis<br><br>UNITED KINGDOM |
| **Corresponding Author Secondary Information:** | |
| **Corresponding Author's Institution:** | |
| **Corresponding Author's Secondary Institution:** | |
| **First Author:** | Mark N. Wass |
| **First Author Secondary Information:** | |
| **Order of Authors:** | Mark N. Wass |
| | Larry Ray |
| | Martin Michaelis |
| **Order of Authors Secondary Information:** | |
| **Additional Information:** | |
| **Question** | **Response** |
| Are you submitting this manuscript to a special series or article collection? | No |
| **Experimental design and statistics** | Yes |

*Powered by Editorial Manager® and ProduXion Manager® from Aries Systems Corporation*

| | |
|---|---|
| Full details of the experimental design and statistical methods used should be given in the Methods section, as detailed in our Minimum Standards Reporting Checklist. Information essential to interpreting the data presented should be made available in the figure legends.<br><br>Have you included all the information requested in your manuscript? | |
| **Resources**<br><br>A description of all resources used, including antibodies, cell lines, animals and software tools, with enough information to allow them to be uniquely identified, should be included in the Methods section. Authors are strongly encouraged to cite Research Resource Identifiers (RRIDs) for antibodies, model organisms and tools, where possible.<br><br>Have you included the information requested as detailed in our Minimum Standards Reporting Checklist? | Yes |
| **Availability of data and materials**<br><br>All datasets and code on which the conclusions of the paper rely must be either included in your submission or deposited in publicly available repositories (where available and ethically appropriate), referencing such data using a unique identifier in the references and in the "Availability of Data and Materials" section of your manuscript.<br><br>Have you have met the above requirement as detailed in our Minimum Standards Reporting Checklist? | Yes |

Manuscript

Click here to download Manuscript Narrative Review_Manuscript.docx

# Understanding of researcher behaviour is required to improve data reliability

Mark N. Wass[1]*, Larry Ray[2], Martin Michaelis[1]*

[1] Industrial Biotechnology Centre and School of Biosciences, University of Kent, Canterbury, UK
[2] School of Social Policy, Sociology and Social Research, University of Kent, Canterbury, UK

E-mail addresses: Mark N. Wass, M.N.Wass@kent.ac.uk; Larry Ray, L.J.Ray@kent.ac.uk; Martin Michaelis, M.Michaelis@kent.ac.uk

*Correspondence to: Mark N. Wass, M.N.Wass@kent.ac.uk; Martin Michaelis, M.Michaelis@kent.ac.uk

**Abstract**

Background: A lack of data reproducibility (“reproducibility crisis”) has been extensively debated across many academic disciplines.

Main body: Although a reproducibility crisis is widely perceived, conclusive data on the scale of the problem and the underlying reasons are largely lacking. The debate is primarily focused on methodological issues. However, examples such as the use of misidentified cell lines illustrate that the availability of reliable methods does not guarantee good practice. Moreover, research is often characterised by a lack of established methods. Despite the crucial importance of researcher conduct, research and conclusive data on the determinants of researcher behaviour are widely missing.

Conclusion: Meta-research is urgently needed that establishes an understanding of the factors that determine researcher behaviour. This knowledge can then be used to implement and iteratively improve measures, which incentivise researchers to apply the highest standards resulting in high quality data.

**Key words**: reproducibility crisis, replication crisis, data reliability, bias, publication bias, meta-research

## Background

A lack of data reproducibility (“reproducibility crisis”) is debated across many medical and scientific disciplines [1-12]. It seems to receive increasing attention as demonstrated by the rise in articles indexed in PubMed [13] related to the terms "reproducibility crisis" and "replication crisis" (Figure 1). This finding is in agreement with another recent analysis that indicated a rapidly increasing number of scientific articles within a “crisis narrative” [14]. Factors suggested to affect reproducibility include (a lack of) methodological standards, (unconscious) bias, pressure related to the need to attract grants and publish in ‘high impact’ journals, and publication bias favouring the publication of novel (“positive”) findings and discouraging the publication of confirmatory findings and “negative” results [3,11,15-22]. Some authors argue that a high proportion (up to 90%) of research money is wasted [2-7], although this very pessimistic view is not unambiguously shared [14]. Nevertheless, the perception of a reproducibility crisis seems to be common among researchers. In two *Nature* surveys, the majority of respondents (52% of 1576 respondents, 86% of 480 respondents) agreed that a reproducibility crisis exists [23,24].

## Main text

### Scale of crisis remains unclear

Despite the high visibility of the issue, systematic research and in turn conclusive evidence on the scale of a potential reproducibility crisis is lacking. In a survey among faculty and trainees at the MD Anderson Cancer Center, about 50% of the participants reported that they had failed to reproduce published data at least once [25]. Similarly, in a *Nature* survey >70% of the 1576 respondents stated that they had been unable to reproduce data at least once [23]. However, systematic data that would enable the reliable quantification of the issue are lacking.

In the “Reproducibility Project: Cancer Biology” by the Center for Open Science [26] and Science Exchange [27], findings from 29 high-profile scientific publications will be independently replicated [28-30]. To date, the results of eleven replication studies have been reported. Important parts of the original paper could be reproduced in four studies [31-34]. The results from two replication studies could not be interpreted [35,36], and two studies failed to replicate the original findings [37,38]. In three further reports, some parts of the original studies were reproduced while others were not [39-41] (Table 1).

Psychological studies also seem to vary with regard to replication success. Very low levels of reproducibility have been reported in some cases [42,43]. A study by the Open Science Collaboration reported the successful replication of 39 of 100 psychological studies [9]. However, other studies replicated a majority of the analysed effects [44] or confirmed previous findings [45,46]. A data set provided a qualitative list of 54 replication attempts of implicit Theory of Mind paradigms based on a survey [47]. 26 studies (48%) were successfully replicated, 15 studies (28%) were partially replicated, and 13 studies (24%) were not successfully replicated [47].

In the clinical research field, an analysis of follow-up publications of 49 original clinical research studies, which had been published between 1990-2003 and had each acquired more than 1000 citations, revealed that seven (16%) were not confirmed by subsequent studies, seven (16%) had reported stronger effects than those found in subsequent studies, 20 (44%) were successfully replicated, and for 11 (24%) follow-up data was not available [1]. Another study compared the results from a limited number of initial clinical studies and respective follow-up studies. It concluded that less than 50% of the investigated studies reported reproducible effects [48]. However, it is not clear how representative the data are.

Notably, reproducibility data has also been reported in articles other than original research articles. For example, researchers from drug companies reported that only six out of 53 studies (11%) [5] or 16 out of 67 studies (24%) [3] had been successfully reproduced. However, these data were published as a Comment [5] and a Correspondence [3] without presentation of detailed data. Hence, the exact nature of the investigations and the criteria for reproducibility remain elusive.

Taken together, there are anecdotal reports of data irreproducibility. However, the actual scale of the issue remains unclear due to a lack of systematic data. Most replication attempts focus on highly cited early-stage studies. This may not adequately reflect the general reproducibility of research findings. A meta-assessment of bias in the sciences observed a significant risk of small, early, and highly cited studies to overestimate effects [49]. Further, failed and successful replication attempts would need to be systematically analysed together to provide meaningful insights. However, such studies are not available. A psychology study estimated that only about 1% of studies are subject to replication attempts [50].

The determination of the scale of the problem may be further complicated by the absence of clear criteria that define the successful or unsuccessful repetition of a study. For example, two large pharmacogenomics screens in cancer cell lines [51,52] provoked a dispute on the consistency of the data, which resulted in at least ten research articles and letters [53-62]. Six of these contributions reported discrepancies between the datasets, while four reported consistency. All six contributions that reported discrepancies were published by the same research group, whereas the articles reporting consistency were published by four different research groups (Table 2). The dispute does not appear to have been resolved. This illustrates that the criteria for reproducibility may differ significantly between researchers. In this context, a modelling study from the psychology field suggests that the criteria for reproducibility may sometimes be interpreted in an unrealistically strict fashion [63].

**Initiatives focus on methodology and data transparency**

The issue of limited reproducibility has also been recognised by research funders and scientific journals [64,65]. For example, the UK funders Medical Research Council, Academy of Medical Sciences, Wellcome Trust, and Biotechnology and Biological Sciences Research Council published a common report on data reproducibility [66] and the World Economic Forum set up a “Code of Ethics for Researchers” [67]. Initiatives to improve data reproducibility typically focus on methodological issues and data transparency. Journals have also tried to address the problem with publishers including the Nature Publishing group and EMBO Press introducing 'publication checklists' [see e.g. 24,68,69]. Nature has also published a special collection on reproducibility in 2013 [70].

**Impact of suggested measures is not clear**

However, limited data are available on the impact of the suggested measures to improve data quality and reproducibility. There are recent reports on shortcomings in data sharing in metabolomic studies [71] and limited adherence to animal reporting guidelines in Korea [72]. A survey reported that psychologists were open to changes to data collection, reporting, and publication practices, but less positive about mandatory conditions of publication [73]. 49% of 480 respondents (out of 5,375 researchers who had published in a Nature journal between July 2016 and March 2017 and who had received the survey) of a Nature survey felt that the checklist had improved the quality of research published in Nature journals [24]. However, it remains unclear if this cohort is representative. One study suggested that reporting of randomisation, blinding, and sample-size estimation in animal experiments had improved in the journal Nature in response to the introduction of the publication checklist based on a comparison of articles published in Nature and Cell from 2013 to 2015 [74]. A preprint posted on bioRxiv also concluded that the introduction of a checklist by Nature had improved study design and the transparency of data [75], but data indicating whether this translated into improved reproducibility are not yet available.

Many authors argue in favour of the standardisation of methods and higher requirements for experimental design [5;18-21,76-78]. In the area of drug discovery, clear requirements for the generation of reproducible data have been suggested [see e.g. 19,21,22,79]. However, data on the implementation of such measures and their efficacy with regard to improved reproducibility are not available. In addition, there is not yet a consensus on the correct methodological approach to achieve high reproducibility. In animal experiments, batch-to-batch variation was described even

under highly standardised conditions in the same lab [80]. In this context, experiment heterogenisation and a multi-laboratory design were suggested to produce more reliable data [80-84] instead of increased standardisation. Notably, standardisation is only an option if the appropriate procedure that delivers correct results is known. Otherwise, a standardised approach may produce flawed results with high reproducibility.

**The availability of appropriate methods does not ensure good practice**

Despite the focus of the debate on research methodology and reporting guidelines, it remains unclear whether (and if yes, to what extent) a lack of reproducibility may be caused by a lack of (knowledge of) appropriate methods and to what extent the significance of data can be improved by tighter guidelines and standardisation.

With regard to the use of appropriate methodologies, cell line misidentification has been an area of concern since the first cell lines were established [85,86]. Although short tandem repeat (STR) analysis has been available and promoted as a reliable authentication method since at least 2001 [87], very recent articles continue to demonstrate that the use of misidentified cell lines remains an issue [88-90]. Similar issues have been reported on the use of antibodies that lack specificity [91-94].

A meta-analysis considering articles published over a 60-year period indicated that the statistical power of behavioural sciences studies has not increased, although the need to increase the statistical power was repeatedly discussed and demonstrated [95]. Hence, the availability of suitable and reliable methods is not sufficient to guarantee their appropriate and consequent use. Additionally, it is often a characteristic of research that both experiments are performed and methodologies are used for the first time. Consequently, researcher conduct and the research culture are critical to ensure the highest possible reliability of data. Accordingly, 82% of the 480

Nature survey respondents felt that researchers have the greatest capacity to improve the reproducibility of published work. 58% thought that individual researchers and 24% thought that laboratory heads were in a crucial position to improve data reliability [24]. Hence, more focus and effort need to be invested to understand how researchers report and present their data and why they do what they do. In this context, 66% of the respondents stated “selective reporting” as a factor that contributes to limited reproducibility [24].

**Role of the incentive system**

Research is performed in a competitive environment. Researchers’ careers are driven by publications in as highly prestigious research journals as possible to gain visibility and attract research funding [19,65,96]. This requires the presentation of novel, significant findings, which incentivises the publication of 'positive' findings and discourages the publication of 'negative' findings. This may also incentivise smaller (potentially underpowered) studies, because they are more likely to produce significant results than larger studies [19,96]. A modelling study indicated that the best strategy to produce significant findings and optimise research output is to perform small studies that only have 10-40% statistical power, which would result in half of the studies reporting false-positive findings [97]. Further, modelling studies suggested that a pressure to produce a high number of outputs with a focus on novel findings and positive results undermines the rigorousness of science, because it leads to a higher proportion of false positives [95,98]. Accordingly, early, highly-cited studies seem to be more likely to present exaggerated findings [49]. However, it remains unclear if (and if yes to what extent) such strategies significantly affect researcher conduct (consciously or subconsciously) and data reproducibility.

**Contribution of publication bias**

A focus on 'positive' results also favours 'publication bias', i.e. 'positive' results are more likely to be published than 'negative' findings. Hence, the available literature does not appropriately represent the totality of experiments that have been performed, because many ‘negative’ results remain unpublished (“file drawer problem”). Additionally, 'positive' findings are more likely to be published in prestigious journals than 'negative’ findings [18,19,99].

One study reported the overestimation of the importance of anticipated prognostic factors in various types of cancer due to publication bias [100]. A follow-up study, which investigated 1,915 research articles on prognostic markers in cancer, found that >90% of studies reported positive prognostic correlations [101]. Less than 1.5% of the investigated articles provided purely ‘negative’ data. Where ‘negative’ findings were presented, this typically happened in the context of other significant correlations (‘positive’ findings), or the authors followed up on non-significant trends and tried to defend the importance of the investigated markers despite the lack of significance [101]. This illustrates that negative results are not commonly published. The evaluation of meta-analyses on cancer biomarkers and the analysis of animal studies on stroke and neurological diseases also suggested a bias towards the publication of ‘positive results’ [102-104].

Further, a similar publication bias was reported for both clinical trials [105,106] and psychological studies [107,108]. A survey-based dataset listed replication attempts of implicit Theory of Mind paradigms. 28 out of the 54 studies, which were reported by the survey respondents, had been published in peer-reviewed scientific journals [47]. The vast majority of published studies (23/ 82%) reported successful replications. Four studies (14%) reported partial replications, and only one study (4%) reported a failed

replication attempt. In sharp contrast, only three of the 26 unpublished replication studies (12%) reported successful replication. Eleven unpublished studies (42%) reported partial replication, while twelve unpublished studies (46%) were unsuccessful replication attempts [47]. Accordingly, a large analysis using US data concluded that there is a general publication bias towards the publication of 'positive' results across the academic disciplines [109]. This bias seems to be more pronounced, the less results are characterised by exact quantitative data [110]. Notably, this topic becomes complicated by findings that suggest that meta-research on publication bias may itself be subject to publication bias [111]. Taken together, there is convincing evidence that a bias favouring the publication of 'positive' findings exists and that it may affect the reliability of publicly available data. However, the scale of the impact is not clear.

**Further determinants of researcher conduct and the impact on data reproducibility are unclear**

Researcher conduct defines the reliability of findings beyond publication bias. This is highly relevant as original research is typically defined by a significant level of novelty in the absence of established standards. Findings are often made using novel (combinations of) approaches together with (novel) model systems and/ or (novel) data for the first time, i.e. before tested and standardised approaches are available. It is fair to think that the incentives provided in a research environment substantially influence researcher behaviour. A substantial meta-analysis based on data from 18 surveys concluded that a pooled weighted estimate of 1.97% (crude unweighted mean: 2.59%) of the respondents admitted to have fabricated, falsified or modified data or results at least once. 14.12% (crude unweighted mean: 16.66%) reported to personally know of a colleague who had done so [112]. Hence, there is evidence of questionable research practices, but the actual extent, the influence of the research

environment and its incentives, and the concrete effect on data reliability remain elusive.

Studies that investigated researcher (mis)conduct in response to the pressures and incentives of the research environment are rare. A survey analysing the answers of 3247 early- and mid-career scientists suggested that a feeling of injustice may contribute to questionable research practices, which may affect reproducibility [113,114]. Focus group discussions involving 51 scientists from research universities revealed that the pressure to produce outputs also promotes questionable research practices [115], which may affect reproducibility. In a survey among 315 Flemish biomedical scientists, 15% of the respondents admitted that they had fabricated, falsified, plagiarised, or manipulated data in the past three years. 72% rated the publication pressure as "too high" [116]. A follow-up qualitative focus group interview study among Dutch biomedical researchers suggested that the current publication culture leads to questionable research practices among junior and senior biomedical scientists [117]. Hence, there is some initial evidence that the pressure associated with a highly competitive environment affects researcher conduct, which in turn affects the reliability and reproducibility of data. Again, however, the actual scale and impact on data reliability remain elusive.

## Conclusions

A reproducibility crisis is widely recognised among researchers from many different fields [23,24]. There is no shortage of suggestions on how data reproducibility could be improved [5,8,11,15-19,21,22,65,68,69,76-79,81,91,107], but quantitative data on the subject (including the scale of the problem) are largely missing. Currently, there is a strong focus on methodology. However, ongoing issues with the use of misidentified cell lines illustrate that problems may persist, despite effective standards being

available. Further, it is in the nature of research to do things for the first time before established methods are available. Hence, data reliability is primarily defined by the conduct of researchers and their rigour and scrutiny in the acquisition, analysis, interpretation, and presentation of data.

Publication bias favours the publication of 'positive' results. Moreover, there are initial indications that the high pressure associated with a competitive environment increases the preparedness of researchers to lower their ethical standards, but the available information remains scarce and the actual impact unclear. Hence, systematic (meta-)research is needed into the topic in order to quantify the issue and generate the knowledge that is necessary to improve data quality and reproducibility. Actual fraud seems to be rare and the exception [14]. Consequently, a major focus of meta-research on data reproducibility will need to be put on researcher behaviour in areas that are not considered to be “fraud” but that still may affect the robustness of data. “Boundary work”, that is, the ways researchers draw the boundaries between the permissible and the non-permissible [118] will be critical here. Only measures that are based on a detailed understanding of researcher behaviour and that are closely monitored for efficacy (and iteratively improved) will make it possible to amend our research system in a way that it provides the right incentives to ensure that researchers apply the highest possible standards and provide high quality data.

**Availability of data and material**

All data are available in the manuscript.

**Competing interest**

There are no competing interests.

**Funding information**

Not applicable

**Authors' contributions**

All authors analysed data, contributed to the writing of the article, and approved the final version.

## References

1) Ioannidis JP. Contradicted and initially stronger effects in highly cited clinical research. JAMA 2005;294:218-28.

2) Young SS, Bang H, Oktay K. Cereal-induced gender selection? Most likely a multiple testing false positive. Proc Biol Sci. 2009;276:1211–2; discussion 1213.

3) Prinz F, Schlange T, Asadullah K. Believe it or not: how much can we rely on published data on potential drug targets? Nat Rev Drug Discov. 2011;10:712.

4) Young SS, Karr A. Deming, data and observational studies: a process out of control and needing fixing. Significance. 2011;9:122-6.

5) Begley CG, Ellis LM. Drug development: Raise standards for preclinical cancer research. Nature 2012;483:531-3.

6) Peers IS, Ceuppens PR, Harbron C. In search of preclinical robustness. Nat Rev Drug Discov. 2012;11:733–4.

7) Young SS, Miller HI. Are medical articles true on health, disease? Sadly, not as often as you might think. Genetic Engineering and Biotechnology News 2014;34:7-9.

8) Begley CG, Buchan AM, Dirnagl U. Robust research: Institutions must do their part for reproducibility. Nature 2015;525:25-7.

9) Open Science Collaboration. Estimating the reproducibility of psychological science. Science 2015;349:aac4716.

10) Kousta S, Ferguson C, Ganley E. Meta-Research: Broadening the Scope of PLOS Biology. PLoS Biol. 2016;14:e1002334.

11) Lilienfeld SO. Psychology's Replication Crisis and the Grant Culture: Righting the Ship. Perspect Psychol Sci. 2017;12:660-4.

12) Hutson M. Artificial intelligence faces reproducibility crisis. Science 2018;359:725-6.

13) https://www.ncbi.nlm.nih.gov/pubmed. Accessed 12 January 2018.

14) Fanelli D. Opinion: Is science really facing a reproducibility crisis, and do we need it to? Proc Natl Acad Sci U S A. 2018;115:2628-2631.

15) Casadevall A, Fang FC. Reforming science: methodological and cultural reforms. Infect Immun. 2012;80:891-6.

16) Fang FC, Casadevall A. Reforming science: structural reforms. Infect Immun. 2012;80:897-901.

17) Ioannidis JP. How to make more published research true. PLoS Med. 2014;11:e1001747.

18) Ioannidis JP, Greenland S, Hlatky MA, Khoury MJ, Macleod MR, Moher D, Schulz KF, Tibshirani R. Increasing value and reducing waste in research design, conduct, and analysis. Lancet 2014;383:166-75.

19) Begley CG, Ioannidis JP. Reproducibility in science: improving the standard for basic and preclinical research. Circ Res. 2015;116:116-26.

20) Jarvis MF, Williams M. Irreproducibility in Preclinical Biomedical Research: Perceptions, Uncertainties, and Knowledge Gaps. Trends Pharmacol Sci. 2016;37:290-302.

21) Kaelin WG Jr. Publish houses of brick, not mansions of straw. Nature 2017;545:387.

22) Kaelin WG Jr. Common pitfalls in preclinical cancer target validation. Nat Rev Cancer. 2017;17:425-40.

23) Baker M. 1,500 scientists lift the lid on reproducibility. Nature 2016;533:452-4.

24) Nature Editorial. Checklists work to improve science. Nature 2018;556:273-4.

25) Mobley A, Linder SK, Braeuer R, Ellis LM, Zwelling L. A survey on data reproducibility in cancer research provides insights into our limited ability to translate findings from the laboratory to the clinic. PLoS One 2013;8(5):e63221.

26) https://cos.io. Accessed on 7 March 2018.

27) https://www.scienceexchange.com. Accessed on 7 March 2018.

28) Errington TM, Iorns E, Gunn W, Tan FE, Lomax J, Nosek BA. An open investigation of the reproducibility of cancer biology research. Elife 2014 Dec 10;3. doi: 10.7554/eLife.04333.

29) Baker M, Dolgin E. Cancer reproducibility project releases first results. Nature 2017;541:269-270.

30) https://elifesciences.org/collections/9b1e83d1/reproducibility-project-cancer-biology. Accessed on 30 October 2018.

31) Aird F, Kandela I, Mantis C; Reproducibility Project: Cancer Biology. Replication Study: BET bromodomain inhibition as a therapeutic strategy to target c-Myc. Elife 2017;6. pii: e21253.

32) Kandela I, Aird F; Reproducibility Project: Cancer Biology. Replication Study: Discovery and preclinical validation of drug indications using compendia of public gene expression data. Elife 2017;6. pii: e17044.

33) Shan X, Fung JJ, Kosaka A, Danet-Desnoyers G; Reproducibility Project: Cancer Biology. Replication Study: Inhibition of BET recruitment to chromatin as an effective treatment for MLL-fusion leukaemia. Elife 2017;6. pii: e25306.

34) Showalter MR, Hatakeyama J, Cajka T, VanderVorst K, Carraway KL, Fiehn O; Reproducibility Project: Cancer Biology. Replication Study: The common feature of leukemia-associated IDH1 and IDH2 mutations is a neomorphic enzyme activity converting alpha-ketoglutarate to 2-hydroxyglutarate. Elife 2017;6. pii: e26030.

35) Horrigan SK; Reproducibility Project: Cancer Biology. Replication Study: The CD47-signal regulatory protein alpha (SIRPa) interaction is a therapeutic target for human solid tumors. Elife 2017;6. pii: e18173.

36) Horrigan SK, Courville P, Sampey D, Zhou F, Cai S; Reproducibility Project: Cancer Biology. Replication Study: Melanoma genome sequencing reveals frequent PREX2 mutations. Elife 2017;6. pii: e21634. doi: 10.7554/eLife.21634.

37) Mantis C, Kandela I, Aird F; Reproducibility Project: Cancer Biology. Replication Study: Coadministration of a tumor-penetrating peptide enhances the efficacy of cancer drugs. Elife 2017;6. pii: e17584. doi: 10.7554/eLife.17584.

38) Repass J; Reproducibility Project: Cancer Biology, Iorns E, Denis A, Williams SR, Perfito N, Errington TM. Replication Study: Fusobacterium nucleatum infection is prevalent in human colorectal carcinoma. Elife 2018;7. pii: e25801.

39) Lewis LM, Edwards MC, Meyers ZR, Talbot CC, Hao H, Blum D, et al. Replication Study: Transcriptional amplification in tumor cells with elevated c-Myc. Elife 2018;7. pii: e30274.

40) Vanden Heuvel JP, Maddox E, Maalouf SW; Reproducibility Project: Cancer Biology, Iorns E, et al. Replication Study: Systematic identification of genomic markers of drug sensitivity in cancer cells. Elife 2018;7. pii: e29747.

41) Eaton K, Pirani A, Snitkin ES; Reproducibility Project: Cancer Biology, Iorns E, Tsui R, et al. Replication Study: Intestinal inflammation targets cancer-inducing activity of the microbiota. Elife 2018;7. pii: e34364.

42) Boekel W, Wagenmakers EJ, Belay L, Verhagen J, Brown S, Forstmann BU. A purely confirmatory replication study of structural brain-behavior correlations. Cortex 2015;66:115-33.

43) Emmerling F, Martijn C, Alberts HJ, Thomson AC, David B, Kessler D, et al. The (non-)replicability of regulatory resource depletion: A field report employing non-invasive brain stimulation. PLoS One 2017;12:e0174331.

44) Klein RA, Ratliff KA, Vianello M, Adams RB Jr Bahník Š, Bernstein MJ, et al. Investigating variation in replicability: A “many labs” replication project. Soc Psychol. 2014;45:142–52.

45) Ahmad MM. Psychometric evaluation of the Cognitive Appraisal of Health Scale with patients with prostate cancer. J Adv Nurs. 2005;49:78-86.

46) Zwaan RA, Pecher D, Paolacci G, Bouwmeester S, Verkoeijen P, Dijkstra K, et al. Participant Nonnaiveté and the reproducibility of cognitive psychology. Psychon Bull Rev. 2018;25:1968-72.

47) Kulke L, Rakoczy H. Implicit Theory of Mind - An overview of current replications and non-replications. Data Brief. 2017;16:101-104. doi: 10.1016/j.dib.2017.11.016

48) Niven DJ, McCormick TJ, Straus SE, Hemmelgarn BR, Jeffs L, Barnes TRM, Stelfox HT. Reproducibility of clinical research in critical care: a scoping review. BMC Med. 2018;16:26.

49) Fanelli D, Costas R, Ioannidis JP. Meta-assessment of bias in science. Proc Natl Acad Sci U S A. 2017;114:3714-9.

50) Makel MC, Plucker JA, Hegarty B. Replications in Psychology Research: How Often Do They Really Occur? Perspect Psychol Sci. 2012;7:537-42.

51) Barretina J, Caponigro G, Stransky N, Venkatesan K, Margolin AA, Kim S, et al. The Cancer Cell Line Encyclopedia enables predictive modelling of anticancer drug sensitivity. Nature. 2012;483:603-7.

52) Garnett MJ, Edelman EJ, Heidorn SJ, Greenman CD, Dastur A, Lau KW, et al. Systematic identification of genomic markers of drug sensitivity in cancer cells. Nature. 2012;483:570-5.

53) Haibe-Kains B, El-Hachem N, Birkbak NJ, Jin AC, Beck AH, Aerts HJ, et al. Inconsistency in large pharmacogenomic studies. Nature 2013;504:389-93.

54) Cancer Cell Line Encyclopedia Consortium; Genomics of Drug Sensitivity in Cancer Consortium. Pharmacogenomic agreement between two cancer cell line data sets. Nature 2015;528:84-7.

55) Bouhaddou M, DiStefano MS, Riesel EA, Carrasco E, Holzapfel HY, Jones DC, et al. Drug response consistency in CCLE and CGP. Nature 2016;540:E9-E10.

56) Geeleher P, Gamazon ER, Seoighe C, Cox NJ, Huang RS. Consistency in large pharmacogenomic studies. Nature 2016;540:E1-E2.

57) Mpindi JP, Yadav B, Östling P, Gautam P, Malani D, Murumägi A, et al. Consistency in drug response profiling. Nature 2016;540:E5-E6.

58) Safikhani Z, El-Hachem N, Smirnov P, Freeman M, Goldenberg A, Birkbak NJ, et al. Safikhani et al. reply. Nature 2016;540:E2-E4.

59) Safikhani Z, El-Hachem N, Smirnov P, Freeman M, Goldenberg A, Birkbak NJ, et al. Safikhani et al. reply. Nature 2016;540:E6-E8.

60) Safikhani Z, El-Hachem N, Smirnov P, Freeman M, Goldenberg A, Birkbak NJ, et al. Safikhani et al. reply. Nature 2016;540:E11-E12.

61) Safikhani Z, El-Hachem N, Quevedo R, Smirnov P, Goldenberg A, Juul Birkbak N, et al. Assessment of pharmacogenomic agreement. F1000Res. 2016;5:825.

62) Safikhani Z, Smirnov P, Freeman M, El-Hachem N, She A, Rene Q, et al. Revisiting inconsistency in large pharmacogenomic studies. Version 3. F1000Res. 2017;5:2333.

63) Stanley DJ, Spence JR. Expectations for Replications: Are Yours Realistic? Perspect Psychol Sci. 2014;9:305-18.

64) Nature Editorial. A code of ethics to get scientists talking. Nature 2018;555:5.

65) Moher D, Naudet F, Cristea IA, Miedema F, Ioannidis JPA, Goodman SN. Assessing scientists for hiring, promotion, and tenure. PLoS Biol. 2018;16:e2004089.

66) https://acmedsci.ac.uk/download?f=file&i=32558. Accessed on 7 March 2018.

67) http://widgets.weforum.org/coe/. Accessed on 7 March 2018.

68) Nature Announcement. Reducing our irreproducibility. Nature 2013;496:398.

69) Nature Editorial. Steps towards transparency in research publishing. Nature 2017;549:431.

70) https://www.nature.com/collections/prbfkwmwvz/. Accessed on 7 March 2018.

71) Spicer RA, Steinbeck C. A lost opportunity for science: journals promote data sharing in metabolomics but do not enforce it. Metabolomics 2018;14:16. doi: 10.1007/s11306-017-1309-5

72) Nam MH, Chun MS, Seong JK, Kim HG. Ensuring reproducibility and ethics in animal experiments reporting in Korea using the ARRIVE guideline. Lab Anim Res. 2018;34:11-19.

73) Fuchs HM, Jenny M, Fiedler S. Psychologists Are Open to Change, yet Wary of Rules. Perspect Psychol Sci. 2012;7:639-42.

74) Han S, Olonisakin TF, Pribis JP, Zupetic J, Yoon JH, Holleran KM, et al. A checklist is associated with increased quality of reporting preclinical biomedical research: A systematic review. PLoS One 2017;12:e0183591.

75) Macleod MR, The NPQIP Collaborative group. Findings of a retrospective, controlled cohort study of the impact of a change in Nature journals' editorial policy for

life sciences research on the completeness of reporting study design and execution. bioRxiv 2017. doi: https://doi.org/10.1101/187245

76) Hatzis C, Bedard PL, Birkbak NJ, Beck AH, Aerts HJ, Stem DF, et al. Enhancing reproducibility in cancer drug screening: how do we move forward? Cancer Res. 2014;74:4016-23.

77) Freedman LP, Cockburn IM, Simcoe TS. The Economics of Reproducibility in Preclinical Research. PLoS Biol. 2015;13:e1002165.

78) Freedman LP, Venugopalan G, Wisman R. Reproducibility2020: Progress and priorities. F1000Res. 2017;6:604.

79) Begley CG. Six red flags for suspect work. Nature 2013;497:433-4.

80) Karp NA, Speak AO, White JK, Adams DJ, Hrabé de Angelis M, Hérault Y, et al. Impact of temporal variation on design and analysis of mouse knockout phenotyping studies. PLoS One 2014;9:e111239.

81) Karp NA. Reproducible preclinical research-Is embracing variability the answer? PLoS Biol. 2018;16:e2005413.

82) Kafkafi N, Golani I, Jaljuli I, Morgan H, Sarig T, Würbel H, et al. Addressing reproducibility in single-laboratory phenotyping experiments. Nat Methods. 2017;14:462-4.

83) Voelkl B, Vogt L, Sena ES, Würbel H. Reproducibility of preclinical animal research improves with heterogeneity of study samples. PLoS Biol. 2018;16:e2003693.

84) Milcu A, Puga-Freitas R, Ellison AM, Blouin M, Scheu S, Freschet GT, et al. Genotypic variability enhances the reproducibility of an ecological study. Nat Ecol Evol. 2018;2:279-2.

85) American Type Culture Collection Standards Development Organization Workgroup ASN-0002. Cell line misidentification: the beginning of the end. Nat Rev Cancer. 2010;10:441-8.

86) Capes-Davis A, Neve RM. Authentication: A Standard Problem or a Problem of Standards? PLoS Biol. 2016;14:e1002477.

87) Masters JR, Thomson JA, Daly-Burns B, Reid YA, Dirks WG, Packer P, et al. Short tandem repeat profiling provides an international reference standard for human cell lines. Proc Natl Acad Sci U S A. 2001;98:8012-7.

88) Vaughan L, Glänzel W, Korch C, Capes-Davis A. Widespread Use of Misidentified Cell Line KB (HeLa): Incorrect Attribution and Its Impact Revealed through Mining the Scientific Literature. Cancer Res. 2017;77:2784-8.

89) Wang M, Yang M, Liu Y, Huang Y, Ye F, Zheng C, Shen C. Investigation of cross-contamination among human cell lines used in China. Int J Cancer. 2017 Aug 10. doi: 10.1002/ijc.30923.

90) Korch C, Hall EM, Dirks WG, Ewing M, Faries M, Varella-Garcia M, et al. Authentication of M14 melanoma cell line proves misidentification of MDA-MB-435 breast cancer cell line. Int J Cancer. 2018;142:561-72.

91) Bradbury A, Plückthun A. Reproducibility: Standardize antibodies used in research. Nature 2015;518:27-9.

92) Uhlen M, Bandrowski A, Carr S, Edwards A, Ellenberg J, Lundberg E, et al. A proposal for validation of antibodies. Nat Methods. 2016;13:823-7.

93) Acharya P, Quinlan A, Neumeister V. The ABCs of finding a good antibody: How to find a good antibody, validate it, and publish meaningful data. F1000Res. 2017;6:851.

94) Edfors F, Hober A, Linderbäck K, Maddalo G, Azimi A, Sivertsson Å, et al. Enhanced validation of antibodies for research applications. Nat Commun. 2018;9:4130.

95) Smaldino PE, McElreath R. The natural selection of bad science. R Soc Open Sci. 2016;3:160384.

96) Brembs B. Prestigious Science Journals Struggle to Reach Even Average Reliability. Front Hum Neurosci. 2018;12:37.

97) Higginson AD, Munafò MR. Current Incentives for Scientists Lead to Underpowered Studies with Erroneous Conclusions. PLoS Biol. 2016;14:e2000995.

98) Grimes DR, Bauch CT, Ioannidis JPA. Modelling science trustworthiness under publish or perish pressure. R Soc Open Sci. 2018;5:171511.

99) Nissen SB, Magidson T, Gross K, Bergstrom CT. Publication bias and the canonization of false facts. Elife. 2016;5. pii: e21451.

100) Kyzas PA, Loizou KT, Ioannidis JP. Selective reporting biases in cancer prognostic factor studies. J Natl Cancer Inst. 2005;97:1043-55.

101) Kyzas PA, Denaxa-Kyza D, Ioannidis JP. Almost all articles on cancer prognostic markers report statistically significant results. Eur J Cancer. 2007;43:2559-79.

102) Tsilidis KK, Papatheodorou SI, Evangelou E, Ioannidis JP. Evaluation of excess statistical significance in meta-analyses of 98 biomarker associations with cancer risk. J Natl Cancer Inst. 2012;104:1867-78.

103) Sena ES, van der Worp HB, Bath PM, Howells DW, Macleod MR. Publication bias in reports of animal stroke studies leads to major overstatement of efficacy. PLoS Biol. 2010;8:e1000344.

104) Tsilidis KK, Panagiotou OA, Sena ES, Aretouli E, Evangelou E, Howells DW, et al. Evaluation of excess significance bias in animal studies of neurological diseases. PLoS Biol. 2013;11:e1001609.

105) Hall R, de Antueno C, Webber A; Canadian Research Ethics Board. Publication bias in the medical literature: a review by a Canadian Research Ethics Board. Can J Anaesth. 2007;54:380-8.

106) Lindner MD, Torralba KD, Khan NA. Scientific productivity: An exploratory study of metrics and incentives. PLoS One 2018;13:e0195321.

107) Bakker M, van Dijk A, Wicherts JM. The Rules of the Game Called Psychological Science. Perspect Psychol Sci. 2012;7:543-54.

108) Ferguson CJ, Heene M. A Vast Graveyard of Undead Theories: Publication Bias and Psychological Science's Aversion to the Null. Perspect Psychol Sci. 2012;7:555-61.

109) Fanelli D. Do pressures to publish increase scientists' bias? An empirical support from US States Data. PLoS One 2010;5:e10271.

110) Fanelli D. "Positive" results increase down the Hierarchy of the Sciences. PLoS One 2010;5:e10068.

111) Dubben HH, Beck-Bornholdt HP. Systematic review of publication bias in studies on publication bias. BMJ. 2005;331:433-4.

112) Fanelli D. How many scientists fabricate and falsify research? A systematic review and meta-analysis of survey data. PLoS One 2009;4:e5738.

113) Martinson BC, Anderson MS, de Vries R. Scientists behaving badly. Nature 2005;435:737-8.

114) Martinson BC, Anderson MS, Crain AL, de Vries R. Scientists' perceptions of organizational justice and self-reported misbehaviors. J Empir Res Hum Res Ethics. 2006;1:51-66.

115) de Vries R, Anderson MS, Martinson BC. Normal Misbehavior: Scientists Talk about the Ethics of Research. J Empir Res Hum Res Ethics. 2006;1:43-50.

116) Tijdink JK, Verbeke R, Smulders YM. Publication pressure and scientific misconduct in medical scientists. J Empir Res Hum Res Ethics. 2014;9:64-71.

117) Tijdink JK, Schipper K, Bouter LM, Maclaine Pont P, de Jonge J, Smulders YM. How do scientists perceive the current publication culture? A qualitative focus group interview study among Dutch biomedical researchers. BMJ Open. 2016;6:e008681.

118) Hesselmann F, Wienefoet V, Reinhart M. Measuring Scientific Misconduct—Lessons from Criminology. Publications 2014;2:61-70.

**Table 1**. Replication studies performed as part of the ‘Replication Project: Cancer Biology’ [30], presented according to the outcome as interpreted in the ‘Editors’ Summary’.

| First author | Title |
|---|---|
| *Editors' Summary: This Replication Study has reproduced important parts of the original paper.* | |
| Irawati Kandela | Replication Study: Discovery and preclinical validation of drug indications using compendia of public gene expression data [32][1] |
| Fraser Aird | Replication Study: BET bromodomain inhibition as a therapeutic strategy to target c-Myc [31] |
| Xiaochuan Shan | Replication Study: Inhibition of BET recruitment to chromatin as an effective treatment for MLL-fusion leukaemia [33] |
| Megan Reed Showalter | Replication Study: The common feature of leukemia-associated IDH1 and IDH2 mutations is a neomorphic enzyme activity converting alpha-ketoglutarate to 2-hydroxyglutarate [34] |
| *Editors' Summary: This Replication Study has reproduced important parts of the original paper, but it also contains results that are not consistent with some parts of the original paper.* | |
| L Michelle Lewis | Replication Study: Transcriptional amplification in tumor cells with elevated c-Myc [39] |
| *Editors' Summary: This Replication Study has reproduced some parts of the original paper but other parts could not be interpreted.* | |
| John P Vanden Heuvel | Replication Study: Systematic identification of genomic markers of drug sensitivity in cancer cells [40] |
| *Editors' Summary: The results in this Replication Study could not be interpreted.* | |
| Stephen K Horrigan | Replication Study: Melanoma genome sequencing reveals frequent PREX2 mutations [36] |
| Stephen K Horrigan | Replication Study: The CD47-signal regulatory protein alpha (SIRPa) interaction is a therapeutic target for human solid tumors [35] |
| *Editors' Summary: This Replication Study has reproduced some parts of the original paper but it also contains results that are not consistent with other parts of the original paper.* | |
| Kathryn Eaton | Replication Study: Intestinal inflammation targets cancer-inducing activity of the microbiota [41] |
| *Editors' Summary: This Replication Study did not reproduce those experiments in the original paper that it attempted to reproduce.* | |
| Christine Mantis | Replication Study: Coadministration of a tumor-penetrating peptide enhances the efficacy of cancer drugs [37] |
| John Repass | Replication Study: Fusobacterium nucleatum infection is prevalent in human colorectal carcinoma [38] |

[1] Number in the reference list

**Table 2**. Articles contributing to a dispute on the consistence of the data derived from two large pharmacogenomic screens [51,52].

| **First author** | **Title** |
|---|---|
| *In favour of consistence* | |
| JP Mpindi | Consistency in drug response profiling. [57] |
| M Bouhaddou | Drug response consistency in CCLE and CGP. [55] |
| P Geeleher | Consistency in large pharmacogenomic studies. [56] |
| Cancer Cell Line Encyclopedia Consortium.; Genomics of Drug Sensitivity in Cancer Consortium. | Pharmacogenomic agreement between two cancer cell line data sets. [54] |
| *In dispute of consistence* | |
| Z. Safikhani | Revisiting inconsistency in large pharmacogenomic studies. [62] |
| Z. Safikhani | Safikhani et al. reply. [58] |
| Z. Safikhani | Safikhani et al. reply. [59] |
| Z. Safikhani | Safikhani et al. reply. [60] |
| Z. Safikhani | Assessment of pharmacogenomic agreement. [61] |
| B Haibe-Kains | Inconsistency in large pharmacogenomic studies. [53] |

**Figures**

**Figure 1**

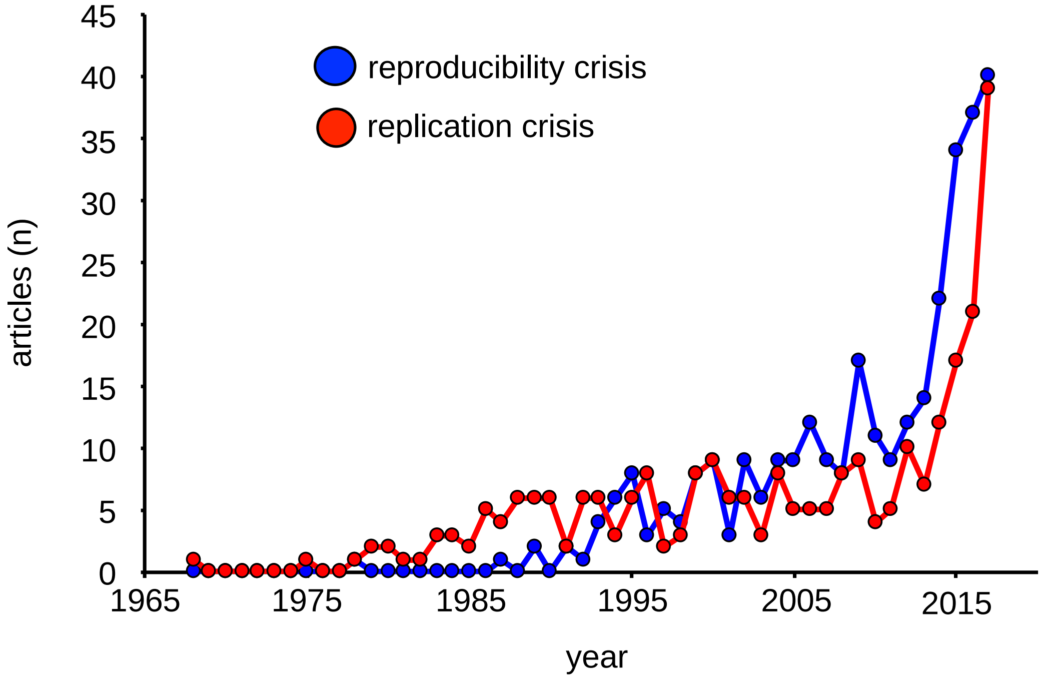


**Figure 1**. Number of articles that are identified by the search terms “replication crisis” (red) or “reproducibility crisis” (blue) per year from 1965 to 2017 in PubMed (www.ncbi.nlm.nih.gov/pubmed, data accessed on 12th January 2018).
