# Supplementary material for: Understanding of researcher behavior is required to improve data reliability
Source: Gigascience. 2019 Jan 31;8(5):giz017. doi: 10.1093/gigascience/giz017 (PMC6528747; doi:10.1093/gigascience/giz017)
Supplement: Response_to_Reviewer_Comments_Original_Submission.pdf [file giz017_response_to_reviewer_comments_original_submission.pdf]

Author's Response To Reviewer Comments

Close

Reviewer #1: The authors discuss the extend the 'reproducibility crisis' is being investigated and what areas of research have been conducted. The authors highlight the need for further investigation to gain a clearer understanding of the scale of these issues, as well as an investigation on the effectiveness of incentives to improve reproducibility of research.

Overall, I thought the review article was clearly written and summarized much of the evidence, highlighting relevant articles where appropriate with a focus on biomedical research, which is appropriate considering the audience of the journal. Below are some suggestions the authors should consider:

1) Not everyone agrees with the term 'reproducibility crisis' and if the narrative is accurate and needed. I suggest the authors consider incorporating these perspectives. Below are perspective articles the authors should consider on this topic:
a. Fanelli, D., 2018. Opinion: Is science really facing a reproducibility crisis, and do we need it to? Proceedings of the National Academy of Sciences 115, 2628-2631. https://doi.org/10.1073/pnas.1708272114
b. Vazire, S., 2018. Implications of the Credibility Revolution for Productivity, Creativity, and Progress. Perspectives on Psychological Science 13, 411-417. https://doi.org/10.1177/1745691617751884

Authors' response:
We have modified the Introduction as follows to illustrate this (p. 3, lines 45-48):
"However, this very pessimistic view may not be widely shared. Other authors argue that the crisis narrative is exaggerated and that periods of self-correction and self-improvement are an immanent feature of scientific research [14,23]."

2) The authors might also want to consider the 'wisdom of the crowd' regarding reproducibility. That is, are researchers able to accurately judge which articles will reproduce? Below are articles the authors should consider on this topic:
a. Benjamin, D., Mandel, D.R., Kimmelman, J., 2017. Can cancer researchers accurately judge whether preclinical reports will reproduce? PLOS Biology 15, e2002212. https://doi.org/10.1371/journal.pbio.2002212
b. Camerer, C.F., Dreber, A., Holzmeister, F., Ho, T.-H., Huber, J., Johannesson, M., Kirchler, M., Nave, G., Nosek, B.A., Pfeiffer, T., Altmejd, A., Buttrick, N., Chan, T., Chen, Y., Forsell, E., Gampa, A., Heikensten, E., Hummer, L., Imai, T., Isaksson, S., Manfredi, D., Rose, J., Wagenmakers, E.-J., Wu, H., 2018. Evaluating the replicability of social science experiments in Nature and Science between 2010 and 2015. Nature Human Behaviour 2, 637-644. https://doi.org/10.1038/s41562-018-0399-z
c. Dreber, A., Pfeiffer, T., Almenberg, J., Isaksson, S., Wilson, B., Chen, Y., Nosek, B.A., Johannesson, M., 2015. Using prediction markets to estimate the reproducibility of scientific research. Proceedings of the National Academy of Sciences 112, 15343-15347. https://doi.org/10.1073/pnas.1516179112

Authors' response:
To address this, the chapter 'Scale of crisis remains unclear' was amended as follows (p. 6, lines 101-107):
"Some studies have investigated the extent to which researchers may be able to estimate the reproducibility of data but conclusive evidence is still missing. Individual cancer researchers were not able to predict accurately whether studies would be reproducible in the "Reproducibility Project: Cancer Biology" [29,52]. However, studies from the social and psychological sciences suggested that the 'wisdom of the crowd' of researchers in the respective fields predicts the reproducibility with higher accuracy than expected by chance [53,54]."

3) The authors discuss three of the four main 'drivers' of the academic research ecosystem: funders,

journals, and researchers. Others have discussed their absence in efforts to improve reproducibility (e.g. Begley et al, 2015). The authors should consider including institutions and researcher training both as part of the ecosystem worth studying, but also how the knowledge meta-research gains can be applied.
a. Begley, C.G., Buchan, A.M., Dirnagl, U., 2015. Robust research: Institutions must do their part for reproducibility. Nature 525, 25-27. https://doi.org/10.1038/525025a

Authors' response:
To address the potential role of institutions and researcher training, we amended the section "Initiatives focus on methodology, data transparency, researcher training, and institutional standards" (title expanded from "Initiatives focus on methodology and data transparency", line 120/121) as follows (p. 7, lines 131/132):
"Moreover, researcher training and institutional standards including quality management systems have been suggested [8,69,75,76]."

Close
