# Supplementary material for: Understanding of researcher behavior is required to improve data reliability
Source: Gigascience. 2019 Jan 31;8(5):giz017. doi: 10.1093/gigascience/giz017 (PMC6528747; doi:10.1093/gigascience/giz017)
Supplement: Reviewer_1_Report_Original_Submission -- Timothy M. Errington, Ph.D. — 12/6/2018 Reviewed [file giz017_reviewer_1_report_original_submission.pdf]

Reviewer Report

Title: Understanding of researcher behaviour is required to improve data reliability

Version: Original Submission **Date:** 12/6/2018

Reviewer name: Timothy M. Errington, Ph.D.

Reviewer Comments to Author:

The authors discuss the extend the 'reproducibility crisis' is being investigated and what areas of research have been conducted. The authors highlight the need for further investigation to gain a clearer understanding of the scale of these issues, as well as an investigation on the effectiveness of incentives to improve reproducibility of research.
Overall, I thought the review article was clearly written and summarized much of the evidence, highlighting relevant articles where appropriate with a focus on biomedical research, which is appropriate considering the audience of the journal. Below are some suggestions the authors should consider:
1) Not everyone agrees with the term 'reproducibility crisis' and if the narrative is accurate and needed. I suggest the authors consider incorporating these perspectives. Below are perspective articles the authors should consider on this topic:
a. Fanelli, D., 2018. Opinion: Is science really facing a reproducibility crisis, and do we need it to? Proceedings of the National Academy of Sciences 115, 2628-2631. https://doi.org/10.1073/pnas.1708272114
b. Vazire, S., 2018. Implications of the Credibility Revolution for Productivity, Creativity, and Progress. Perspectives on Psychological Science 13, 411-417. https://doi.org/10.1177/1745691617751884
2) The authors might also want to consider the 'wisdom of the crowd' regarding reproducibility. That is, are researchers able to accurately judge which articles will reproduce? Below are articles the authors should consider on this topic:
a. Benjamin, D., Mandel, D.R., Kimmelman, J., 2017. Can cancer researchers accurately judge whether preclinical reports will reproduce? PLOS Biology 15, e2002212. https://doi.org/10.1371/journal.pbio.2002212
b. Camerer, C.F., Dreber, A., Holzmeister, F., Ho, T.-H., Huber, J., Johannesson, M., Kirchler, M., Nave, G., Nosek, B.A., Pfeiffer, T., Altmejd, A., Buttrick, N., Chan, T., Chen, Y., Forsell, E., Gampa, A., Heikensten, E., Hummer, L., Imai, T., Isaksson, S., Manfredi, D., Rose, J., Wagenmakers, E.-J., Wu, H., 2018. Evaluating the replicability of social science experiments in Nature and Science between 2010 and 2015. Nature Human Behaviour 2, 637-644. https://doi.org/10.1038/s41562-018-0399-z
c. Dreber, A., Pfeiffer, T., Almenberg, J., Isaksson, S., Wilson, B., Chen, Y., Nosek, B.A., Johannesson, M., 2015. Using prediction markets to estimate the reproducibility of scientific research. Proceedings of the National Academy of Sciences 112, 15343-15347. https://doi.org/10.1073/pnas.1516179112
3) The authors discuss three of the four main 'drivers' of the academic research ecosystem: funders, journals, and researchers. Others have discussed their absence in efforts to improve reproducibility (e.g. Begley et al, 2015). The authors should consider including institutions and researcher training both as

part of the ecosystem worth studying, but also how the knowledge meta-research gains can be applied.
a. Begley, C.G., Buchan, A.M., Dirnagl, U., 2015. Robust research: Institutions must do their part for reproducibility. Nature 525, 25-27. https://doi.org/10.1038/525025a

Methods

Are the methods appropriate to the aims of the study, are they well described, and are necessary controls included? Choose an item.

Conclusions

Are the conclusions adequately supported by the data shown? Choose an item.

Reporting Standards

Does the manuscript adhere to the journal's guidelines on minimum standards of reporting? Choose an item.

Choose an item.

Statistics

Are you able to assess all statistics in the manuscript, including the appropriateness of statistical tests used? Choose an item.

Quality of Written English

Please indicate the quality of language in the manuscript: Choose an item.

Declaration of Competing Interests

Please complete a declaration of competing interests, considering the following questions:

- Have you in the past five years received reimbursements, fees, funding, or salary from an organisation that may in any way gain or lose financially from the publication of this manuscript, either now or in the future?
- Do you hold any stocks or shares in an organisation that may in any way gain or lose financially from the publication of this manuscript, either now or in the future?
- Do you hold or are you currently applying for any patents relating to the content of the manuscript?
- Have you received reimbursements, fees, funding, or salary from an organization that holds or has applied for patents relating to the content of the manuscript?
- Do you have any other financial competing interests?
- Do you have any non-financial competing interests in relation to this paper?

If you can answer no to all of the above, write 'I declare that I have no competing interests' below. If your reply is yes to any, please give details below.

I declare that I have no competing interests

I agree to the open peer review policy of the journal. I understand that my name will be included on my report to the authors and, if the manuscript is accepted for publication, my named report including any attachments I upload will be posted on the website along with the authors' responses. I agree for my report to be made available under an Open Access Creative Commons CC-BY license (http://creativecommons.org/licenses/by/4.0/). I understand that any comments which I do not wish to be included in my named report can be included as confidential comments to the editors, which will not be published.

I agree to the open peer review policy of the journal

To further support our reviewers, we have joined with Publons, where you can gain additional credit to further highlight your hard work (see: https://publons.com/journal/530/gigascience). On publication of this paper, your review will be automatically added to Publons, you can then choose whether or not to claim your Publons credit. I understand this statement.

Yes Choose an item.
